# COVID-19 lockdown and natural resources: a global assessment on the challenges, opportunities, and the way forward

**DOI:** 10.1186/s42269-022-00706-2

**Published:** 2022-01-29

**Authors:** Meseret Muche, Getahun Yemata, Eyayu Molla, A. Muthama Muasya, Berhanu Abraha Tsegay

**Affiliations:** 1 Department of Biology, Woldia University, P.O. Box 400, Woldia, Ethiopia; 2 Department of Biology, Bahir Dar University, Bahir Dar, Ethiopia; 3 Deparment of Natural Resource Management, College of Agriculture and Environmental Sciences, Bahir Dar University, Bahir Dar, Ethiopia; 4 Department of Biological Sciences, University of Cape Town, Rondebosch, 7700 South Africa

**Keywords:** Biodiversity, COVID-19, Environmental resources, Global gasses, Lockdown

## Abstract

**Background:**

The Coronavirus (COVID-19) is a global pandemic caused by SARS-CoV-2, which has an enormous effect on human lives and the global environment. This review aimed to assess the global scientific evidence on the impact of COVID-19 lockdown on natural resources using international databases and search engines. Thus, the unprecedented anthropause due to COVID-19 has positive and negative effects on natural resources.

**Main body:**

This review showed that the unprecedented pandemic lockdown events brought a negative impact on the physical environment, including pollution associated with a drastic increase in person protective equipment, deforestation, illegal poaching and logging, overfishing, disruption of the conservation program and projects. It is noted that the spread of pandemic diseases could be aggravated by environmental pollution and a rapid increase in the global population. Despite these negative impacts of COVID-19, the anthropause appear to have also several positive effects on natural resources such as short term reduction of indoor and outdoor environmental pollutants (PM2.5, PM10, NO_2_, SO_2_, CO, and CO_2_), reduction in noise pollutions from ships, boats, vehicles, and planes which have positive effects on aquatic ecosystems, water quality, birds behaviour, wildlife biodiversity, and ecosystem restoration.

**Conclusion:**

Therefore, governments and scientific communities across the globe have called for a green recovery to COVID-19 and implement multi-actor interventions and environmentally friendly technologies to improve and safeguard sustainable environmental and biodiversity management and halt the next pandemic.

## Background

In December 2019 in Wuhan city of China, a novel coronavirus (SARS-CoV-2; severe acute respiratory syndrome) has garnered global attention due to its rapid transmission (Chauhan 2020; WHO 2020a). The World Health Organization (WHO) declared as a new coronavirus (COVID-19) and a global pandemic disease on 11 March 2020 after phylogenetic studies with SARS viruses (WHO 2020b). Since then the disease (COVID-19) has spread to different countries all over the world leading to an ongoing pandemic (Khan et al. 2021). The COVID-19 pandemic is an unprecedented, wreaking havoc, and the latest episode in a string of environment-borne human tragedies, catastrophic in its magnitude and repercussions (Bang and Khadakkar 2020). Since February 2020, many countries have a lockdown decrees to restrict the movement of their citizens in their cities, industries, public transportation, and closed international and interstate borders (Khan et al. 2021; Praveena and Aris 2021). These lockdown measures to control the impact of COVID-19 have caused an unforeseen reduction in global economic, social, and transport activities, and the physical environment (Bar 2020; Corlett et al. 2020; Rume and Islam 2020; Venter et al. 2020; Gkatzelis et al. 2021). Reports of several countries have indicated that the COVID-19 lockdown events caused huge socio-economic disruption, which brought positive and negative impacts on the natural environment (Bar 2020; Akinsorotan et al. 2021; McNeely 2021; Praveena and Aris 2021). Some of the positive effects include reduction of air pollutants and land surface temperature, slowdown of discharging industrial effluents, and reduction of noise (Rume and Islam 2020; Khan et al. 2021; McNeely 2021). Also, quarantine actions brought negative influences on natural resource management such as a drastic increase in plastic and medical wastes, deforestation in the tropics, disruption of the conservation program and projects, etc. (Bates et al. 2020; Brancalion et al. 2020; Decaro et al. 2020; Saadat et al. 2020; Akinsorotan et al. 2021; Filho et al. 2021; Praveena and Aris 2021; Razanatsoa et al. 2021). Therefore, this review paper aimed to provide a wide-ranging insight into the global impacts (positive and negative) of COVID-19 lockdown decrees on natural resources.

## Review methodology

**Fig. 1:**
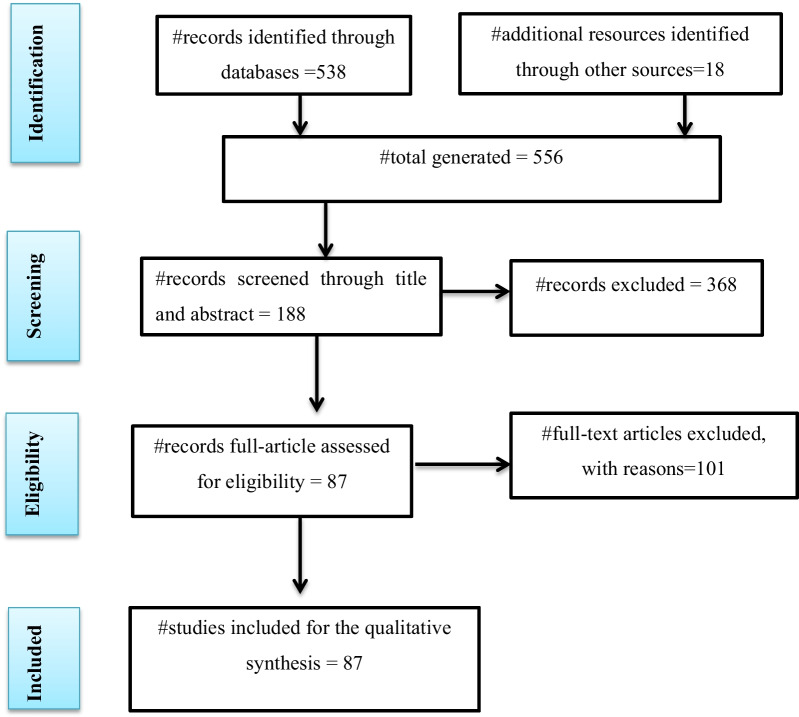
Flowchart for selecting eligible studies on the challenges and opportunities of COVID-19 lockdown on natural resources

The methodological approach adopted in this review was a broader literature search and synthesis of relevant peer-reviewed articles. The research articles related to the impact of COVID-19 lockdown on ranges of natural resource management systems were systematically assessed. International databases and platforms of PubMed, Web of Science, Scopus, Google Scholar, and reports were searched for English articles published between 11 March 2020 (COVID-19 lockdown started) and June 30, 2021. The review process was further complemented by searching the relevant articles using the following themes: (TITLE-ABS KEY('environment' OR 'biodiversity' OR 'pollution' OR 'water ANDquality' OR 'air ANDquality')) AND(TITLE-ABS-KEY ('AND COVID-19 ANDlockdown'))). To manage and remove duplicates, all searched literatures were imported to EndNote X7 software (Thompson Reuter, CA, USA). Initially, in this review, studies not suitable for the outcome of interest were excluded. Accordingly, eligible studies were screened to synthesize knowledge and experiences based on the inclusion criteria and then 87 publications were included (Fig. 1). The review primarily presented a comprehensive overview of the cause, transmission, and health impact of COVID-19. Accordingly, based on empirical data sources and opinions, the negative and positive influences of the COVID-19 lockdown on the natural ecosystems were critically assessed, with particular emphasis on the aquatic ecosystem (water quality and aquatic life), terrestrial ecosystem (soil, vegetation, and wildlife), and the policy measures and interventions for future natural resource managements to block the ongoing impact of the virus and other related zoonotic disease outbreaks. At last, concluding remarks and future perspectives were drawn on the sustainable management of natural resources.

### Comprehensive overview of COVID-19

Coronaviruses are important human and animal pathogens (Chauhan 2020) that can cause illnesses, such as the common cold, severe acute respiratory syndrome (SARS), and Middle East respiratory syndrome (MERS) (Zhou et al. 2020; Rana et al. 2021). Coronavirus disease 2019 (COVID-19) is caused by severe acute respiratory syndrome coronavirus 2 (SARS-CoV-2). It's an enveloped, positive-sense, and single-stranded RNA virus belonging to the β-coronavirus subfamilies (Afewerky 2020; Hu et al. 2020; Zhu et al. 2020a, b; Kumar et al. 2021). SARS-CoV-2 has a diameter of 60 nm to 140 nm and distinctive spikes, ranging from 9 to 12 nm, giving the virions the appearance of a solar corona (Wiersinga et al. 2020). Phylogenetic studies revealed that the SARS-CoV-2 genome sequence is closely related to bat-SL-CoVZC45 and bat-SLCoVZXC21 with 96% identity (Chauhan 2020; Lu et al. 2020; Zhou et al. 2020). However, SARS-CoV-2 shares only 40% sequence identity with MERS-CoV (Hu et al. 2020). And therefore, it appears likely that SARS-CoV-2 has been naturally evolved from bats (Zhou et al. 2020; Rana et al. 2021), but whether the COVID-19 virus is transmitted directly from bats is unknown (Perlman 2020). The process of SARS-CoV and SARS-CoV-2 coronaviruses' entrance into the host is facilitated by the host cells Transmembrane protease serine 2 (TMPRSS2) and lysosomal proteases (Zou et al. 2020). The TMPRSS2 promotes viral uptake by cleaving angiotensin-converting enzyme 2 (ACE2) and activating the SARS-CoV-2 spike glycoproteins, which mediates coronavirus entry into host cells (Hu et al. 2020; Wiersinga et al. 2020). Together the presence of the ACE-2 receptor and TMPRSS2 in the target cell determines the host susceptibility to the SARS-CoV-2 virus (Afewerky 2020).

SARS-CoV-2 is spread primarily via saliva and respiratory droplets during direct person-to-person contact while coughing, sneezing, talking, or singing (Karia et al. 2020; WHO 2020d; Wiersinga et al. 2020). Infection might also occur if a person's hands are contaminated by these secretions or by touching contaminated surfaces and then touching the surface of eyes, nose, or mouth (WHO 2020d; Rana et al. 2021). Yet, there is no evidence that animals play a significant role in spreading SARS-CoV-2 (CDC 2021). The average time from exposure to symptom onset is five days, and 97.5% of people who develop symptoms do so within 11.5 days (Wiersinga et al. 2020). The severity of COVID-19 symptoms can range from very mild to severe, and thus the most common symptoms are fever, dry cough, myalgia, fatigue, and lower respiratory signs (Afewerky 2020; Hu et al. 2020; Perlman 2020). Some people may experience worsened symptoms, such as worsened shortness of breath and pneumonia, about a week after symptoms start (Wiersinga et al. 2020). As far as clinical manifestations are concerned, this particular virus has exhibited deleterious impacts on systems other than the respiratory system (primary target organ), such as the brain, hematological system, liver, kidneys, and endocrine system (Rana et al. 2021). Therefore, to combat the impact of COVID-19, wash hands frequently, maintain social distancing, practice respiratory hygiene, and when fever, coughs, and difficulty breathing, seek medical care early. Moreover, the serologic test and everyone ages 16 and older can get a COVID-19 vaccine booster shot to increase the immune response against a part of the virus called the spike protein.

## Main text

### The nexus between COVID-19 lockdown and natural resources

#### Spill-over effect on soil ecology and vegetation biodiversity

**Fig. 2:**
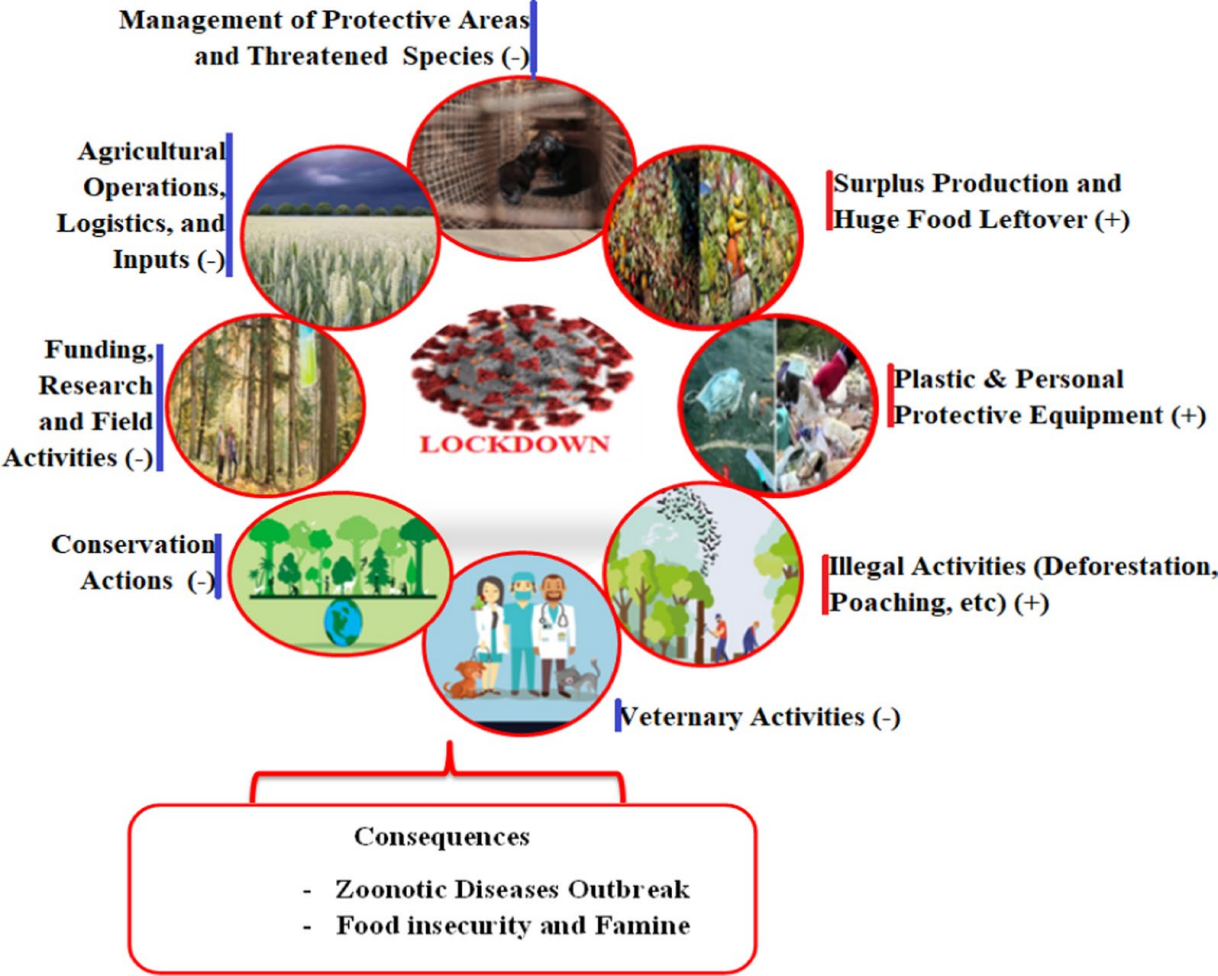
The negative effect of COVID-19 lockdown on environmental management and biodiversity management operations, (**+**) indicates increasing trends and (**−**) reduction in activities

Living organisms depend on the existence of healthy soils for the production of food, ensuring a bio-diverse environment, and underpinning the current and future human health, well-being, and economic prosperity (Bang and Khadakkar 2020; OECD 2020a; Poch et al. 2020). The ongoing COVID-19 lockdown measures, while useful in preventing COVID-19 spread, have seriously affected the global environment due to socio-economic disruptions and the migration of labourers from the urban that shifted their activities and pressure on natural resources (FAO 2020a; Akinsorotan et al. 2021). In particular, the agricultural and biodiversity sectors related to soil, forest resources, livestock, water, fisheries, and wildlife management have been the most affected areas (FAO 2020a; Hockings et al. 2020; Lal et al. 2020; Poch et al. 2020). The direct influences of the COVID-19 pandemic on soil and related components are through human activities, such as a decline in human consumption give rise to surplus food that is being disposed of and added to the soil (Lal et al. 2020; Fig. 2). For instance, mass burials of swine and poultry in the US; and a potato glut in French fries, dumping millions of gallons of milk per day. Moreover, the impacts have been observed on beef producers during the COVID-19 pandemic border closures, lockdowns, and curfews (Lal et al. 2020; Kumar et al. 2021). This has long-term consequences on the soil, groundwater quality, and biodiversity (Rume and Islam 2020).

As the huge organic food leftovers are decomposed, they release global gases such as CO_2_, CH_4_, and N_2_O, which would be contributing to the formation of carbonic acid that ultimately reduces soil and water pH (Lal et al. 2020). Besides, the lockdowns and curfews within the country and across borders have threatened the agricultural systems and specifically the soil quality by interrupting agro-processing and logistics. People are unable to manage the farm operations, such as tilling, hoeing, harvesting, and conserving, getting access to agricultural inputs (e.g. seeds, fertilizers), and increasing post-harvest loss (FAO 2020b; Poch et al. 2020; Fig. 2). Consequently, soil degradation rates increased and the spreading of invasive alien species by interrupting the controlling measures, which ultimately brings a serious of impacts on environmental sustainability (Arora et al. 2020; Bang and Khadakkar 2020; FAO 2020b; Praveena and Aris 2021). Soil degradation, in turn, increases the atmospheric CO_2_ emissions, which contribute to climate change (Poch et al. 2020) and significantly amplified the risk of zoonoses (Arora et al. 2020; Poch et al. 2020). Also, worldwide usage of COVID-19 pandemic personal protective equipments (e.g., gloves, protective medical suits, masks, etc.), hand sanitizers, antimicrobial soaps, and use of mass disinfectants, such as sodium hypochlorite, hypochlorous acids, and chlorine have brought a severe threat to soil ecosystem (Rume and Islam 2020; Zhu et al. 2020a, b; Benson et al. 2021; Khan et al. 2021; Klemes et al. 2020; Silva et al. 2021; Fig. 2). For instance, sodium hypochlorite used for mopping floors, offices, rooms, hospitals for killing the virus has a very toxic effect on the environment and microorganisms in the soil systems (Atolani et al. 2020). Similarly, the alcohol-containing products and detergents spilled in the water are toxic to aquatic fauna and flora. Spill in soil may also pollute the groundwater (Rume and Islam 2020; Kumar et al. 2021).

Forest biodiversity mitigates climate change by removing about a third of the global greenhouse gas emissions each year (Global Forest Watch 2021). However, the large human lockdown to control the COVID-19 pandemic has provided the opportunity and impelled illegal activities such as logging activity and deforestation (Brancalion et al. 2020). The coronavirus infection period also hindered environmental diplomacy efforts and forestry sector field operations in terms of the plantation, afforestation, reforestation, and reduced monitoring by legal enforcement (Mongabay 2020). Tropical forests have suffered the most, with some of the highest rates of forest clearances in Brazil, Colombia, Cambodia, Indonesia, Nepal, and Madagascar, with more anecdotal reports emerging from Myanmar and Peru since the start of the COVID-19 pandemic (UN/DESA 2021). Evidence suggests that a total of 9583 km^2^ (12%) primary forests in global tropics were found to be degraded during the COVID-19 lockdown events, which is nearly double that of 2019 (4732 km^2^) and showing an increasing trend (Brancalion et al. 2020; Global Forest Watch 2021; Fig. 2). For instance, in Brazil, satellite imagery has shown deforestation rates reached 72% between August 2019 and May 2020 compared to baseline levels (Global Forest Watch 2021).

The Amazon rainforest has lost more than 9000 km^2^ (3500 square miles) during the year to March 2020, constituting an increase of 47% and 9.5% compared to 2018 and 2019, respectively. This is the highest annual recorded loss since 2008 (Akinsorotan et al. 2021; Silva-Junior et al. 2021). In addition, increasing rates of forest clearing to make way for oil palm plantations in Indonesia, a higher risk of forest fires in Colombia, and illegal extraction, either timber or other forest products in Nepal have been observed during the COVID 19 lockdown and movement restriction (Mongabay 2020; Global Forest Watch 2021). Within Africa, in West Africa and Madagascar, the COVID-19 pandemic has affected the protected areas because of increasing human pressures due to migrant workers returning from the cities to their local residences and a reduction in field activities of the NGOs’ and state agencies’ to have made forest patrols more challenging (Mongabay 2020). The spread of novel infectious diseases like COVID-19 is an outcome of deforestation, habitat fragmentation, intensive farming, and a growing global population (Arora et al. 2020; Fig. 2). Therefore, sustainable management of forest resources is very vital to deprive future zoonotic disease outbreaks.

##### The lockdown impact on the wildlife and aquatic organisms

The emergence of the COVID-19 pandemic has been affecting wild animal populations and habitats through multiple pathways (Lindsey et al. 2020; Vanapalli et al. 2021). One way is through infecting other species of animals, such as cattle, mice, masked palm civets, bats, dogs, and camels. They have shown symptoms such as dry cough and loss of appetite (Lu et al. 2020). A COVID-19 infected tiger was also reported at New York’s Bronx Zoo (Bar 2020). Accordingly, the lockdown has weakened institutional support for conservation by interrupting funding streams, eroding the protection of parks and vulnerable species, and forestalling vital monitoring and research activities that make these impacts visible (Lindsey et al. 2020; Fig. 2). Moreover, the reductions in law enforcement and human presence in protected areas have contributed to a rise in illegal activities like logging and hunting (Humphrey 2020; McNeely 2021; Vanapalli et al. 2021; Fig. 2). The illegal harvest of wildlife, including rare and threatened species, is increasing in Botswana; for example, both African species of rhinoceros, (*Diceros bicornis* and *Ceratotherium simum*) have been poached to meet the demand for rhino horn to treat the COVID-19 virus in traditional Chinese medicine (Somerville 2020). In Madagascar, the poaching of endangered green turtles has increased, and seventeen rhinos and two elephants have been killed in Namibian in 2020 compared to the past two years (Mupatsi 2020). When logging occurs, animals are forced into different or smaller areas because of wildlife habitats degradation and are more likely to become stressed or sick and eventually led to close contact with people and domestic animals, which is a major driving force for the transmission of disease (Bang and Khadakkar 2020; Akinsorotan et al. 2021; McNeely 2021). The immediate lockdown would also avert some of the veterinary activities regarding preventive vaccination against pre-existing diseases (Gortázar and de la Fuente 2020). In the aquatic ecosystem, centuries-old coral reefs in the Caribbean are irreversibly damaged due to fungal diseases and invasive species such as rats; destroying native species (Bang and Khadakkar 2020). In both Argentina and Indonesia, for example, there are reports of heightened illegal fishing activity by foreign vessels, as government priorities have shifted toward pandemic control (Bennett et al. 2020). Similarly, heavy fishing activity has also been reported in Spain and Italy during the lockdown, with reductions reaching up to 50% compared to previous years (Global Fishing Watch 2020).

Reports further have indicated a plethora of negative environmental impacts attributed to wildlife trafficking, increased incidents of human-elephant conflicts, poor sanitation methods in housing and slaughter, and over-fishing due to restricted domestic activities (Bang and Khadakkar 2020; Bar 2020; McNeely 2021). Changes in feeding sites and the formation of new competitive systems in synanthropic species suddenly deprived of anthropogenic food (Łopucki et al. 2021). Also, the negative perception of wildlife as disease carriers may result in retaliatory killing of possible carriers species such as bats and pangolins, resulting in severe repercussions for these threatened species (Bang and Khadakkar 2020; Vanapalli et al. 2021). Moreover, disturbance of ecological processes has resulted in an abundance of “generalist” or “opportunistic” species. This increase creates a higher potential for the occurrence of effective zoonotic hosts and increases the risk of a future disease outbreak and nearly 50% of zoonotic diseases that have emerged in humans are associated with ecosystem degradation (OECD 2020a). These biodiversity perils lose the wildlife buffering zones, and hence bringing domestic animals and people close to pathogen-carrying wildlife. The upcoming recession driven by the COVID-19 pandemic may also increase poverty and food insecurity in deforestation frontiers, leading to greater bush-meat consumption and increased chances of new zoonotic diseases (Brancalion et al. 2020; Fig. 2). Therefore, we asserted that during this time, safeguarding and sustainable management of natural resources and biodiversity by maintaining the ecological integrity will be dwindled the vulnerability and steer clear of the upcoming pandemic and other zoonotic disease outbreaks.

## COVID-19 lockdown: optimisms to air quality, water quality & terrestrial ecosystems

### Air quality and its impact on the global environment

**Fig. 3:**
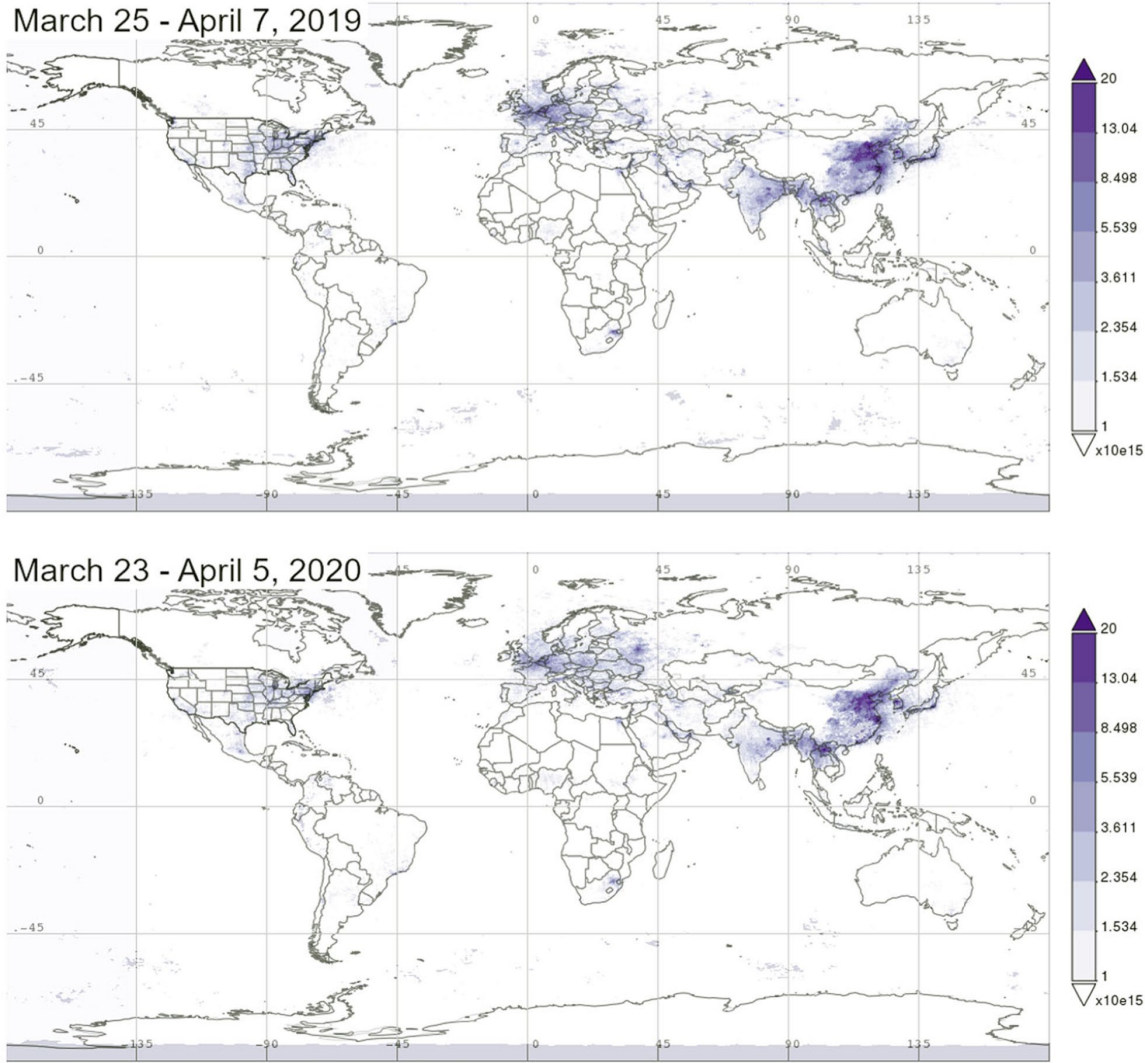
Nitrogen dioxide (NO_2_) levels in the troposphere before and after anthropause period. For example, the NO_2_ content in Madrid, Milan and Rome decreased by about 45%, and by 54% in Paris, compared to 2019. From Copernicus Sentinel data 2019–2020 processed by KNMI/ESA (Sources: ESA 2020; NASA 2020)

**Fig. 4:**
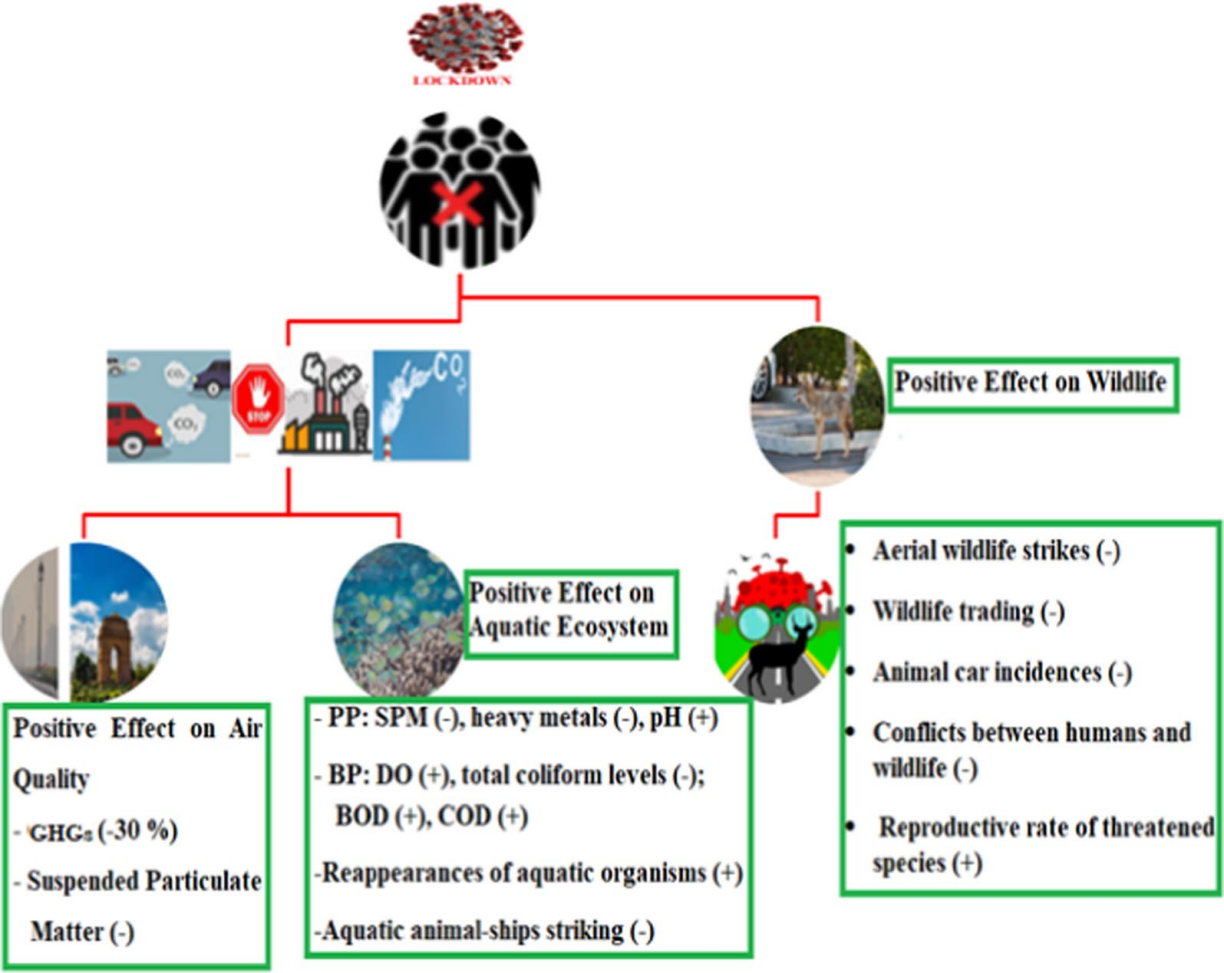
The positive effect of COVID-19 lockdown on environmental management and biodiversity management operations, (**+**) indicates increasing trends and (**−**) reduction. GHGs; Greenhouse Gases; PP, Physical Parameter; SPM, Suspended Particulate Matter; pH, Power of Hydrogen; BP, Biological Parameter; DO, Dissolved Oxygen; BOD, Biological Oxygen Demand; COD, Chemical Oxygen Demand

In many countries, economic growth has exacerbated air pollutant emissions with severe consequences for the environment and human health (Venter et al. 2020). The sources of pollutants include the transport sectors, industries, power generation stations, residential energy use, and tourism sectors (Rume and Islam 2020). Fine particulate matter with a diameter less than 2.5 and 10 μm (PM2.5 and PM10), nitrogen oxide (NO_2_), methane (CH_4_), sulfur oxide (SO_2_), black carbon (BC), carbon monoxide (CO), and ozone (O_3_) are the principal constituents of air pollutants (Saadat et al. 2020; Bhat et al. 2021; Khan et al. 2021; Kumar et al. 2021). Of these, the most prevalent threats to human health are PM2.5 and PM10 (Khan et al. 2021). A survey in the US revealed that an increased exposure of only 1 μg/m^3^ to PM2.5 is associated with an 8% increase in the COVID-19 mortality rate (Wu et al. 2020). Outdoor air pollution is estimated to cause the death of 7 million people annually (WHO 2020c). After the lockdown to control COVID-19 expansion, the major ambient (outdoor) air pollution sources have almost been halted (Muhammad et al. 2020; Nakada and Urban 2020; Rume and Islam 2020; Venter et al. 2020). Reports from all over the world have indicated that the reduction of human activities has provided an inadvertent benefit for the earth. This has lessened the anthropogenic intervention on qualitative degradation of environmental components at very local to global scales (Mandal and Pal 2020; Rupani et al. 2020; Bhat et al. 2021; Praveena and Aris, 2021). Thus, environmental conditions including air and water qualities have been improved due to lockdown events (Lokhandwala and Gautam 2020; Figs. 3; 4). For instance, studies showed that the COVID-19 lockdown across countries in the world resulted in a 30% decrease in air pollutants while mobility was curbed by approximately 90% (Muhammad et al. 2020; Kumar et al. 2021). A marginal increase in ozone has been reported (Venter et al. 2020). Adams (2020), Nakada and Urban (2020), Rume and Islam (2020), and Bhat et al. (2021) have reviewed that strong quarantine is estimated to reduce the emission of NO_2_ and CO by nearly 25.5% in the US. Specifically, NO_2_ levels have been decreased by 31%, 25%, 16%, and 10% in Los Angeles, San Diego, Phoenix, and Las Vegas, respectively, compared to previous years (OMI 2020). In New York, the level of air quality has been improved by almost 50% due to measures taken to restrict the spread of the virus (Saadat et al. 2020).

The troposphere satellite data taken in 34 countries during COVID-19 lockdown indicates that the concentration of nitrogen dioxide (NO_2_) and particulate matter levels (PM2.5) have been reduced by 60% and 31%, respectively (Venter et al. 2020). The highest reductions of NO_2_ (20–40%) and PMs (5–15%) pollutants have been reported in France, Portugal, Finland, Norway, Poland, Sweden, and Spain (ESA 2020; Muhammad et al. 2020; NASA 2020; Rupani et al. 2020; Skiriene and Stasiškiene 2021; Fig. 3). Increases in O_3_ concentrations have also been observed: 50% in Barcelona, 24% in Nice, 14% in Rome, 11% in London, and 2.4% in Valencia compared to the same period in 2017–2019 (Sicard et al. 2020; Mousazadeh et al. 2021). This could be due to the unprecedented reduction in NO*x* emissions, leading to a lower O_3_ titration by the NO (Mousazadeh et al. 2021). Studies have confirmed that COVID-19 lockdown events have led to air pollution reduction across China, where NO_2_, PM10, PM2.5, and CO decreased by 33.1–37.8%, 33.6%, 7.4–21.5%, and 12.7–20.4%, respectively (Wang et al. 2021). On average, 11.4% air quality improvement has been reported (Saadat et al. 2020). Sharma et al. (2020) have reported that a − 51.84, − 53.11, − 17.97, − 52.68, − 30.35, 0.78, and − 12.33% in the levels of PM10, PM2.5, SO_2_, NO_2_, CO, O_3_, and NH_3_, respectively in India within the period of precautionary strategies to prevent COVID-19 transmission. Similar drastic reductions in NO (− 77.3%), NO_2_ (− 54.3%), and CO (− 64.8%) concentrations have been observed in Brazil during partial lockdown compared to the pre-lockdown (Bhat et al. 2021). A significant reduction in PM2.5, PM10, NO_2_, and CO air pollutants levels has resulted due to the COVID-19 pandemic in Korea (Ju et al. 2021). In Bangladesh, the NO_2_ content dropped down up to 45–54%, and the suspended particulate matter (SPM) and carbon emission decreased by 7% due to restrictions in transportation sectors and causes the improvement in water and air qualities (Bar 2020).

In Africa, the CO_2_ emission levels have decreased between 0.01 and 0.03 mol/m^2^, except in West Africa with 0.04 mol/m^2^. In some areas, the emission is higher than 0.05 mol/m^2^ where indoor air pollution has increased as people are confined to their homes (Mupatsi 2020). In Egypt, the COVID-19 pandemic has had a great effect in reducing the air pollution levels of NO_2_, O_3_, PMs, CO, and greenhouse gas emissions (Mostafa et al. 2021). Similarly, high air quality was reported in Morocco (Cherif et al. 2020), Sudan, and Madagascar (Mupatsi 2020), but, the impact of the pandemic on air pollution is not yet reported in Ethiopia, despite a declaration of a two weeks lockdown restriction in the country and then continued vehicle mobility and economic activities. Therefore, the effects of COVID-19 lockdowns on the environment have necessitated a much demanding transformational change to safeguard environmental sustainability across the world. As a result, the sight of the blue sky created a sense of optimism among the people towards a clean and better environment. Enhancing environmental health through better air quality will reduce the vulnerability to pandemics and improve overall societal well-being and resilience (OECD 2020b).

### Impact on water quality and aquatic life

Aquatic ecosystems support a substantial pool of the earth’s biological diversity, such as microorganisms, invertebrates, insects, plants, and fish. Yet, for decades, the hydrosphere has been severely polluted because of rapid urbanization, industrialization, and overexploitation (Rume and Islam 2020; Yunus et al. 2020). However, many studies from all over the world have reported that COVID-19-induced lockdown has had beneficiary consequences to the improvement of surface water quality and quantity in some rivers, canals, and seas (Yunus et al. 2020; Khan et al. 2021; Mousazadeh et al. 2021; Tokatl and Varol 2021; Table 1; Fig. 4). For instance, 40–50% overall water quality improvement of the Ganga river in India (the most polluted river in the world) has been observed and met drinking water standards in just two months lockdown period (Khan et al. 2021; Mousazadeh et al. 2021).

**Table 1:** Exemplary of the effect of COVID-19 lockdown on water quality parameters

Country	River/Canals/Seas	Water quality parameters	Lockdown period	Before lockdown period	After lockdown period	Change (After to before lockdown)	References
India	Ganga river	DO	–	2.3 mg/L	4.8 mg/L	2.5 mg/L	Khan et al. (2021)
	Sabarmati River	TUR	24-Feb-2020 to 4-May-20	12.72 mg/L	8.08 mg/L	− 4.64 mg/L	Aman et al. (2020)
	Varanasi's Nagwa Nala	DO	March 6 to April 4 2020	3.8 mg/L	6.8 mg/L	3.0 mg/L	Arora et al. (2020)
	Vembanad lake	SPM	28th February 2020 to 16th April 2020	14.4 mg/L	12.1 mg/L	− 2.3 mg/L	Yunus et al. (2020)
Turkey	Meriç-Ergene River Basin (Ergene River and Çorlu Stream)	DO	Mid- January 2020 to early June 2020	8.9 mg/L	6.65 mg/L	− 2.25 mg/L	Tokatl and Varol (2021)
		pH	,,	8.96	8.32	− 0.64	
		TUR	,,	22.46NTU	38.82NTU	16.42 NTU	
		TSS	,,	34.08 mg/L	49.16 mg/L	15.08 mg/L	
		COD	,,	32.82 mg/L	34.33 mg/L	1.52 mg/L	
		BOD	,,	8.23 mg/L	9.22 mg/L	0.96 mg/L	
Northeast Italy	Venice’s canals	TSM	One month	3 g/m3	1.4 g/m3	− 1.6 mg/L	Niroumand-Jadidi et al. (2020)
China	Yangtze River Delta	WQI	March 2019–April 2020	66.0%	77.4%	11.4%	Liu et al. (2021)
Bhutan-India-Bangladesh Trans-boundary rivers	Mahananda (Siliguri st-1)	pH	Pre-lockdown (December 2019 and January 2020) and lockdown (April and May 2020)	6.85	7.44	0.59	Sarkar et al. (2021)
		TUR	,,	17.95 NTU	11.05 NTU	− 6.9 NTU	
		COD	,,	52 mg/L	26 mg/L	-26 mg/L	
		BOD	,,	1.5 mg/L	2.05 mg/L	0.55 mg/L	
		TDS	,,	90 mg/L	96 mg/L	6.0 mg/L	
		DO	,,	6.85 mg/L	7.15 mg/L	0.3 mg/L	
		TSS	,,	56 mg/L	56 mg/L	0	

BOD, Biological Oxygen Demand; COD, Chemical Oxygen Demand; TUR, Turbidity; DO, Dissolved Oxygen; NTU, Nephelometric Turbidity Units; pH, Power of Hydrogen; SPM, Suspended Particulate Matter; TSM, Total Suspended Matter; TSS, Total

Suspended Solids; WQI, Water Quality Index; TDS, Total Dissolved Solids

Landsat-8 operational land imager (OLI) analysis demonstrated that suspended particulate matter (SPM) decreased by 15.9% on average (range: − 10.3% to 36.4%, up to 8 mg/L decrease) in Vembanad Lake, the longest freshwater lake in India compared to the pre-lockdown period (Saadat et al. 2020; Yunus et al. 2020; Kumar et al. 2021). The DO at Varanasi's Nagwa Nala has significantly improved and showed a 79% improvement in concentration (Arora et al. 2020; Table 1). Niroumand-Jadidi et al. (2020) reported that the satellite images analysis in Venice’s canals in northeast Italy has indicated an almost 50% reduction in the total suspended matter (TSM) after one month of lockdown (Table 1). Apparently, water flowing in the channels of Venice became clean than before and the fish have been seen once again in the canals (Niroumand-Jadidi et al. 2020; Saadat et al. 2020; Bhat et al. 2021; Kumar et al. 2021). In Turkey, the heavy metal pollution index, total hazard index values for children and adults, and total carcinogenic risk values for As (60% reduction), Cr (decreased by 94%), Pb and Cd have shown a significant improvement in water quality in the most polluted rivers of the Ergene River and Çorlu stream in Meriç-Ergene River Basin (Tokatl and Varol 2021; Table 1). In the same study, however, no considerable variation has been observed between the pre-lockdown and lockdown periods in terms of key water quality parameters (BOD, COD, EC, turbidity, TSS, and Mn) due to the ongoing domestic wastewater discharges (Table 1). The COVID-19 lockdown improved river water quality index (WQI) by reducing industrial sewage discharges, and thus, WQI of most of the stations shows around 75.9% and 80% improvement of water quality during lockdown period in Yangtze river delta in China and Bhutan-India-Bangladesh trans-boundary River, respectively (Liu et al. 2021; Sarkar et al. 2021; Table 1).

Water pollutions have also been reduced in the beach areas of Bangladesh, Malaysia, Thailand, Maldives, Indonesia, Acapulco (Mexico), and Barcelona (Spain) (Rume and Islam 2020; Mousazadeh et al. 2021). Khan et al. (2021) have reviewed that in Saudi Arabia, the lockdown between February and June or July hastened the recovery of fish stocks and other marine organisms due to reduction in pollution, noise pollution from shipping, and commercial fishing. Also, the Grand Canal of Italy has turned clear, and many aquatic species have reappeared (Niroumand-Jadidi et al. 2020). This astonishing water quality enhancement has been attributed to a reduction in the discharging of industrial effluents into surface waters, tourist banning and a reduction in boat traffic activity. Because of the restriction, ship striking and injuring or killing marine animals, sediment churning, noise, and other water pollutants have been reduced (Saadat et al. 2020; Yunus et al. 2020; Kumar et al. 2021; Mousazadeh et al. 2021; Tokatl and Varol 2021). This supports other creatures such as fish, dolphins, and swans to come back to the canals, seas, and streams (Mousazadeh et al. 2021).

### Effects on terrestrial ecosystems

In recent years, the increase in extreme environmental and weather events, such as pollutions, biodiversity degradations, habitat fragmentations, and pronounced dry spells have already had a massive impact on different aspects of soil, agriculture, forestry, and animal ecology (Lal et al. 2020). Under this scenario, the global COVID-19 pandemic lockdown and restrictions have shown notable environmental changes and dramatically reduced the impacts of anthropogenic disturbances on ecosystems worldwide, the so-called ‘anthropause’ (Zuluaga et al. 2021). Such rapid and widespread changes in the lives of people all over the world must have an impact on the biodiversity of the terrestrial ecosystems (Łopucki et al. 2021; Fig. 4). For example, in the US, the 10-week lockdown (March 25 to June 7, 2020) has led to a 61% reduction in the number of aerial wildlife strikes compared to the previous year (Zuluaga et al. 2021). Bar (2020) has found out that the reduction in noise and pollutant gases in India changes the behaviour of birds, in which the partial migratory birds’ species like open bill stork, painted stork, grey heron, spoonbill, spot-billed pelican, and ibis generally extend and stay a bit longer in the sanctuaries. According to Łopucki et al. (2021), hedgehog roadkill levels during the lockdown have been over 50% lower than in the pre-pandemic years. China has temporarily and other countries permanently have banned wildlife trading during the time of the pandemic, which is a serious threat to biodiversity, where animals such as civets, bats, pangolin, etc. are supposed to be agents for the transmission (Chakrabarty and Maity 2020). This will be good for wildlife health and restoring the sustainable ecosystem. Substantial reductions in vegetation fire count in the order of 5–75% and night-time cooling of land surface temperature (LST) have been observed from space-based data in the majority of Indian states during the fire-prone period, March–April, of 2020 as compared to mean from previous years’ (Lele et al. 2021). The same study has also shown an increase in primary productivity to the tune of a maximum of 38% over Indian forests. These ecological shifts following anthropause are usually viewed as positive and result in biodiversity recovery, fewer conflicts between humans and wildlife, higher reproductive output of threatened species, and improved ecosystem conditions and services (Lal et al. 2020). Therefore, managing the ecosystem process by maintaining the soil, vegetation, and biodiversity of life is of great importance to obtain the proper ecosystem services and goods.

## Conclusions

Rapid increases in the world’s human population from around one billion two centuries ago to over 7.8 billion today (OECD 2020a), has meant more encroachment and poorly managed natural resources and ecosystems. This has brought humans and animals into ever-closer contact and increased the risk of zoonotic disease transmission and would have cataclysmic effects on environmental sustainability. For this reason, environmentalists and ecologists have awakened about the fate of people across the globe, as continuing to exacerbate habitat fragmentation and species loss, pollutions, illegal logging, wildfires, storms, pests, climate change, and subsequent social and economic distortion of people in biodiversity-rich countries. Yet actually, the global COVID-19 crisis forces urgency to address short-term issues, especially in the natural resource management such as air and water qualities, and wildlife biodiversity with some challenges like impeded conservation activities, and encourage illegal actions, etc. Current reports also have shown that as countries begin to ease lockdown events to avert economic collapse, global gas emissions will again rise. Thus, the anthropause response to COVID-19 should pave the way for the implementation of a similar campaign in responses to global environmental and ecological crises that need urgent attention and then design short and long-term strategies, including:

- Implement and promote sustainable natural resource management approaches including implementation of the UN strategic plan for a green COVID-19 recovery 2030, by reversing the loss of forest cover worldwide through sustainable forest management, including protection, restoration, afforestation, reforestation, and increase efforts to prevent forest degradation and contribute to the global effort of addressing climate change.
- Strengthen and effective implementation of policy arena and proclamations related to natural resource management to halt forest loss and fragmentation, improper resource utilization and management practices, deforestation, illegal logging, and illegal wildlife trade, which could help to reduce human-wild animal interactions and evade future zoonotic disease outbreaks.
- Launch forest conservation, sustainable use, and restoration programs and underpin forest and wildlife management sectors, which can able to address eco-friendly COVID-19 prevention, functioning as enhancement of ecosystem services and goods and hence alleviate poverty among rural communities through job and other societal benefits.
- Establishment of the conservation taskforces with the inclusion of local people and their traditional ecological knowledge (TEK), planning and implementing conservation-related research activities to improve the sustainability of the people and environmental management.
- Enhance law enforcement and government interventions in sanctuaries, protected areas, and beaches to minimize the human pressure (visitors) that has a significant contribution to the occurrence of environmental pollutions.

## Data Availability

Not applicable.

## Abbreviations

BOD: Biological oxygen demand
CDC: Centres for disease control and prevention
COD: Chemical oxygen demand
DO: Dissolved oxygen
PM: Particulate matter
NASA: National Aeronautics and Space Administration
NTU: Nephelometric turbidity units
OECD: Organisation for Economic Co-operation and Development
OMI: Ozone monitoring instrument
SPM: Suspended particulate matter
TDS: Total dissolved solids
TSM: Total suspended matter
TSS: Total suspended solids
WQI: Water quality index
UN/DESA: United Nation/Department of Economics and Social Affairs
